# Designing Evidence-Based Family Planning Programs for the Marginalized Community: An Example of Muslim Community in Nepal

**DOI:** 10.3389/fpubh.2016.00122

**Published:** 2016-06-14

**Authors:** Diksha Sapkota, Shiva Raj Adhikari, Tara Bajracharya, Vishnu Prasad Sapkota

**Affiliations:** ^1^Kathmandu University School of Medical Sciences, Dhulikhel, Kavre, Nepal; ^2^Institute for Nepal Environment and Health System Development, Kathmandu, Nepal; ^3^Department of Economics, Tribhuvan University, Kirtipur, Nepal; ^4^Save the Children, Kathmandu, Nepal

**Keywords:** designing program, family planning, mixed method, Muslim community, Nepal

## Abstract

**Context:**

Family planning (FP), considered as an encouraging trend for development, is thought to be positively correlated with family health and well-being and negatively correlated with poverty levels. Despite being a priority goal of government and development agencies, in a heterogeneous society like Nepal, FP can be an issue that needs to be dealt with consideration for religious and cultural beliefs of different sections of society. Despite steady progress in achieving FP goals, minority populations have lagged behind the rest of the country in achieving improved family health outcomes; Muslim community being one such example.

**Objectives:**

This study aims to explore the existing situation of FP use in Muslim communities and to identify key policy-related issues affecting the access to and utilization of FP services.

**Settings and design:**

Mixed approach was used in Kapilbastu district, which accommodated the larger proportion of Muslims in Nepal.

**Materials and methods:**

Interview was conducted among 160 married women using semi-structured questionnaire. Focus group discussion, key informant interviews, and consultative meeting were the qualitative techniques employed in this study. Quantitative data were analyzed using descriptive and inferential statistics (Chi-square test), while qualitative data by thematic approach.

**Results:**

More than half of women (56.0%) expressed their interest in FP use, while reported users were just below the quarter (24.0%). Husband approval and secrecy of their personal identity affect use of any method of contraception. Future plan for children and prior information regarding FP found to affect current use of FP, significantly. FP word itself was found to be stigmatizing, so women prefer replacing the word FP with culturally appropriate one. Furthermore, incorporating it into comprehensive package for improving women’s health will definitely contribute to improve access and uptake of services.

**Conclusion:**

Discrepancy exists between current use and desire for use of FP among Muslim women in future. This highlights the inadequacy of implementing the current blanket policy and programs related to FP and offer ways to move forward with the national FP agenda ensuring the cultural rights and non-discrimination of women.

## Introduction

Nepal is a diversified and pluralistic country in terms of ethnic, linguistic, and religious composition. Different groups of populations speak different languages, practice diverse cultures, and follow different religions (1). As reported in census 2011, currently, there are altogether 125 caste/ethnic groups, 123 languages are spoken as mother tongues, and 10 different types of religions are followed across country (2). On account for such population dynamics, it should be noted that prevailing practices may discriminate individuals or group of that community, and hence, development policies of the country must be based on the notion of “pluralism.” It recognizes the uniqueness and prevailing values of each community and creates an enabling environment, whereby diverse religions, cultures, and traditions can coexist in a nation. Through development policies, the country should recognize individual and collective rights to practice their own religion and culture. However, it is challenging to accommodate diverse social, cultural, ethnic, and religious groups on an equal footing (1, 3). Most of the studies have focused largely on factors influencing contraceptive use, but limited studies have addressed the inadequacy in policy to ensure improved access and utilization of family planning (FP) services (1, 4).

Family planning policy of Nepal was implemented in 1958. It was initially organized around vertical structures with central management and logistics and later integrated into other health programs like Expanded Program on Immunization (EPI), human immunodeficiency virus/sexually transmitted infections (HIV/STIs) (5), etc. Historically, most government planning models and decision-making process have been supply driven, and less emphasis has been given to reducing demand side barriers like distance, opportunity cost, and cultural and social practices. The large gap in access to health services and substantial differences in health indicators across income and ethnic groups are well established across countries all over the world (6). Despite the remarkable progress in FP programs, unmet need is still at 27% in Nepal (7). Additionally, there is a wide variation in the utilization pattern of FP services across different ethnic groups and geographical regions (8). Marginalized communities include those people who face systemic and structural discrimination in the society, which pushed them to the margins. Muslim community is one such community which is considered a religious minority and socially excluded group in Nepal consisting 4.4% of total population (2, 3). According to national level data, although 50% of currently married women were reportedly using FP methods, only 17% of rural Muslim women were found to have used any kind of contraceptive methods (7–9).

Similar to other societies, religious orientation and culture have played a crucial role in shaping peoples’ attitudes within the Muslim community. Among other determinants, behavioral norms prevailing in the Islamic society may be a contributing factor toward hindering the access and utilization of FP services for Muslim women (10). Resistance to FP is driven by several factors ranging from method-specific side effects, opposition from family members, psychological factors, and educational level of couple to religious and political factors (4, 11), etc. In addition to this, there is a common concern in Muslim communities that FP is deemed to be as Western ideology or “conspiracy,” which aims to limit the size of the Muslim population (12). Several studies have shown that people held general consensus that large families were good, as it ensures large number of productive member who can contribute for economic upliftment of the family and, furthermore, it is in line with Islamic teaching (4, 13). However, contraception has a wide range of positive impacts on both the health of women and children and socioeconomic development. It has particularly striking impact on lives of women specifically on survival and empowerment (14).

Developing targeted policies with recognition of religious and cultural rights of the marginalized community and ways to protect women from discriminating traditional practices is a challenging task. Social inclusion and equality in society are equally important. No doubt, there are both opportunities and challenges to develop the policy that ensure the recognition of religious and cultural rights of the marginalized community and protect the women from discriminating traditional practices. It may not be cost-effective to create segregated policy, but policy meant for people is vital for acceptance and achievement of desired goal. The primary question for the policy makers is how to design such policy and programs ensuring its effective implementation. This paper seeks to provide answer of the question with example of policy and programs of FP in the Muslim community of Nepal.

### Current National FP Policy and Programs

Family planning is one of the priority programs of the Government of Nepal (GON) and is considered as a component of reproductive health package and essential health-care services of Nepal Health Sector Program II (2010–2015) (5). The main objective of FP program is to improve the health status and overall quality of whole family by increasing the access and utilization of FP services. For this, various modern contraceptive methods are being provided under national health-care delivery system. FP information and services are provided through government, social marketing, non-governmental organizations, and private sectors. In government setting, temporary FP methods, like male condoms, pills, and injectables, are provided on regular basis through sub health posts (SHPs), health posts (HP), and primary health care (PHC) outreach clinics, and services of implant and intrauterine contraceptive devices (IUCDs) are provided from PHCs and HP where trained personnel are available. Female community heath volunteers (FCHVs) provide information to community people and distribute condoms and pills. Sterilization services are being provided at static sites or through scheduled “seasonal” or mobile outreach services (5). Over the last few decades, Nepal has made steady progress in FP as evidenced by reduction in total fertility rate (TFR) from 5.1 in 1991 to 2.6 in 2011, and improvement in contraceptive prevalence rate (CPR) from 28.5% in 1996 to 49.7% in 2011 (8). Existing FP policies focus on satisfying demand for quality FP services, increasing its accessibility and availability, reducing unmet need, and informed choice, *inter alia*. Despite the high importance placed on FP activities, we are still lagging behind the target, and there are gross disparities in achievement across several ethnic groups. According to Nepal Household Survey 2012, compared with Brahmin/Chhetri ethnic group, CPR among married Muslim women is remarkably low (46 and 13.1%, respectively) (15). Recent national representative survey report showed that poverty incidence increases monotonically with household size and number of children. In a household where there is no any children, poverty is 12%, while it shot up to 47% where there are three or more children (16). Hence, it is justifiable to promote FP programs in our context. However, focus should be shifted from reducing family size to improvement of women’s and child health, as unmet need for FP can lead to unintended pregnancies posing risks for women, their families, and societies (11). However, there are deficits in the literature which captures the information regarding community influences on contraceptive behavior of Muslim communities.

This study aims to explore the existing FP use among Muslim women and identify the factors which influence access to and uptake of FP services. It seeks to assess the gaps in current policy and intends to provide recommendations for solving identified key issues, which will ultimately lead to better policy formulation and program implementation.

## Materials and Methods

### Study Design

Mixed methods approach was used to identify problems of current policy and to improve uptake of and access to FP services in Muslim communities of Kapilbastu district. Kapilbastu district located in the Western Development Region of Nepal was selected as the study site, since it is one of the districts with highest number of Muslim Population (18.2%), and it was feasible to conduct the study being one of the program district of Save the Children, Nepal (17).

### Sample Size for Quantitative Data

A total of 160 married Muslim women of reproductive age group were included in the study, purposively considering the time and financial limitations and sufficient for applying inferential statistics.

### Data Collection Tools and Techniques

Formative assessment was done to obtain the baseline information about the Muslim community and to finalize the villages to be selected for the study. Four village development committees (VDCs) were selected randomly out of 11 VDCs having highest number of Muslims in Kapilbastu district. Out of these four VDCs, two were randomly selected for household survey. As the list of Muslim household number and/or list of married Muslim women of reproductive age group (15–49 years) were not available, it was not possible to prepare the sampling frame of the Muslim household, restricting the use of random sampling technique for household selection. Households were visited and screened for Muslim residence, and the presence of married women of reproductive age group. Data collection proceeded if respondents met the above mentioned criteria, otherwise next household was visited.

A total of four focus group discussions (FGDs) were conducted in four villages selected randomly. Two FGDs were conducted with female participants and two with male participants. Participants included women of reproductive age group who were willing to participate in this study and belonged to Muslim religion. Similarly, married and unmarried Muslim males were included in the focus group discussion. Women who were included in FGDs were not included in the household interview. A consultative meeting with representatives from government and non-government organizations working in the field of FP was organized and 12 key informant interviews (KII) were conducted. Data were collected from both primary and secondary sources. FGDs, each constituting 8–10 participants, were conducted by researcher with the support of other team members in natural setting, in local dress, and in local language. In case of language barriers, field researchers translated the questions in their local language (Awadi), and answers were then re-translated into Nepali to be understood by the team members. Focus group discussion mainly focused on general information regarding FP, reasons behind preferences and non-usage of any FP methods, major constraints in access and utilization of services, recommendations to improve access, use of FP services, etc. Guides for KIIs and consultative meeting also included the factors affecting use and non-use of FP services, difficulties with existing FP policies, potential approaches for improving uptake of FP services, etc.

Quantitative data were collected from household survey *via* interview using semi-structured questionnaire by local enumerators consisting of three females and one male. Local inhabitants belonging to Muslim ethnicity were recruited on the basis of their educational background, knowledge of local language, and prior experience to such studies for quantitative data collection. Sociodemographic information of the respondents was assessed, and questionnaire was designed in such a way to assess the demand side and supply side factors affecting FP use. Demand side factors included current use of FP methods, reasons for not using any devices, community-related factors like discussion of FP use in family, decision makers, attitudes toward FP use, etc. However, in supply side-related questions, access to and satisfaction with the health services were included.

### Quality Assurance

Total 3 days training (both field based and office based) was given to the enumerators to orient them about the study purpose, methodologies, and techniques of data collection. Quantitative and qualitative tools were designed in such a way that they complemented each other.

Pretesting was conducted among 10% of the sample and adjustments were made accordingly. Subject experts were consulted for content validity. Mock FGDs were conducted to validate the questions. Collected data were checked thoroughly on the day of data collection, and in case of gross errors, enumerators were instructed to revisit the respondents and recollect data. Field activities were supervised and monitored by researcher herself. Double entry was done, and data were analyzed by a trained data analyst. A number of quality check mechanisms were used to detect and minimize errors during the data entry stage. Ethical clearance was obtained from Nepal Health Research Council (NHRC).

### Data Management and Analysis

Master chart was prepared in Microsoft Excel for quantitative data. Data were then transferred into Statistical Package for Social Sciences (SPSS) Version 20, and descriptive data were presented in frequencies and percentages. Bivariate analysis (chi-square test) was done due to categorical nature of data and level of significance set was 0.05. Odds ratio (OR) was calculated from bivariate analysis and was presented with 95% confidence interval value. For qualitative analysis, transcription was done and Nepali transcription was translated into English by researchers. Open coding was done by two researchers separately, and inter-coder agreement was assessed. Themes were generated, and it was finalized after subsequent discussion with the research team, and key findings were presented accordingly. An integration of qualitative and quantitative data provides both general and in-depth information for the study. Quantitative and qualitative data were triangulated and validated as per the model suggested by Steckler et al. (18).

## Results

Findings cover FP-related issues at individual, family, and societal levels. The study has looked into its current use, decision-making process, and factors that determine use and non-use of FP services. Key policy-related issues have also been explored. Convergent parallel design was used, where results from quantitative and qualitative approach were compared or related to look for patterns or contradictions (19). Methodological triangulation was done by adopting mix methods to yield mutual validation and convergence of the result.

### Sociodemographic Findings

Out of 160 respondents, majority (42.5%) were of age group 20–30 years. One in 10 women was in her teen age. Average age of the respondents was about 30 years. Almost two-third women had never attended any formal education, and very few (less than 10%) had received modern education. Nearly nine in ten women were housewives followed by laborers. Husbands of nearly half of the respondents had attended modern education system, and nearly one-third did not have any formal education. Majority of the husbands were foreign labor migrants (43.1%), followed by farmers. Almost 60% of the respondents had more than three children and on an average, a woman had four children (Table 1).

**Table 1:** **Sociodemographic characteristics of respondents (*n* = 160)**.

Background variables	Categories	Frequency (%)
Age of mothers in years	<20 years	16 (10.0)
	20–30 years	68 (42.5)
	30–40 years	58 (36.3)
	>40 years	18 (11.3)
	Mean (SD)	31.68 (0.61)
Respondent’s education	Modern and madarasa education	8 (5.0)
	Modern education only	6 (3.8)
	Madarasa only	51 (31.9)
	No formal education	95 (59.4)
Respondent’s occupation	Housewife	139 (86.9)
	Labor	20 (12.5)
	Business	1 (0.6)
Husband’s education	Modern and madarasa education	39 (24.4)
	Modern education only	42 (26.3)
	Madarasa only	29 (18.1)
	No formal education	50 (31.3)
Husband’s occupation	Agriculture	44 (27.5)
	Labor	31 (19.4)
	Foreign migrant workers	69 (43.1)
	Others	16 (10.0)
Number of children	Equal to or less than 3 children	69 (43.1)
	More than 3 children	91 (56.9)

*Laborers include those who are involved in daily wage-based work, but are working in their own country, and foreign labor migrants include those who have been out of their country of origin for employment*.

In FGDs, women of reproductive age group were included, and it was ensured that both married and unmarried women were present to collect the diverse views regarding FP. Similarly in male group, adolescents, adults, and elderly were included. Involving them helped to collect information about their expectations from FP-related policies and analyze successes and failures of existing one.

### Family Planning: From Societal and Individual Perspective

We can’t use FP methods as we have no any right to prevent a new life from coming to the world. [KII with female religious leader, Taulihawa]

It is the law of nature and every child is born with his/her fate. Allah will take care of his children, so it is not a problem [KII with male youth from Bedauli]

Children are the gift of Allah, and according to our hadith, we cannot decide on the number of children. But if there is a medical problem, we can use FP methods [KII with female maulana (religious leader), Taulihawa]

In Quran, it was clearly mentioned that we can stop child bearing in case of any type of risks, be it medical, financial, or social risk. [male FGD participant, Bedauli]

Every society has its values and beliefs, which play an influential role in the access and uptake of services. It was found that Muslims had different religious interpretations regarding the use of FP services, which can be classified into two schools of thought: one group openly accepts and promotes the use of contraception considering it as need of changing times while another group strongly opposes it with the belief that, except for medical reasons, contraception is a violation of societal values.

More children are reason for more trouble; I even plead Allah for not giving me many children. [female FGD participant, Gauri VDC]

We all feel that having small number of children is good. But we don’t have options; we can’t express our feelings. [female FGD participant, Maharagjunj VDC]

Earlier, we did not have options, except giving as many births as we can. But now, I even advise my daughter-in-law to use FP methods. [mother-in-law, FGD at Gauri VDC]

Between these two extremes, there were people who relate their attitudes and behavior regarding FP to their religion, but also believe that large family creates troubles.

### Family Planning and Health: Interrelated Concept

I wouldn’t have looked this old if I had less number of children; my health has deteriorated significantly compared to what it used to be. (sighs) [a middle aged woman, FGD in Gauri VDC]

I have three sons, so each will get a smaller portion of paternal property. She has only one son; hence, they are financially stronger. [Female FGD participant, Gauri VDC]

According to the views of most of the women who participated in the FGDs, good health was desired by all and women expressed that having more children was reason for ill-health and premature aging.

### Social Secrecy and Spousal Influence in FP Use

Allah knows everything; nothing is hidden from him. But we cannot openly share the use of FP services as others will see it negatively. [a newly married woman, FGD Gauri VDC]

I use Depo and my husband knows about this, but my in-laws don’t. It is not necessary for them to know as we live separately [married young female at FGD, Gauri VDC]

People wanted secrecy in their FP use as they were bound within societal norms and values where they had to display such behaviors which were deemed acceptable for all in community.

When women were asked about the major influencer for FP-related decision-making, significantly larger proportion responded that it was their husbands (72.5%) followed by oneself.

Both qualitative and quantitative findings showed that husband played key role in decision-making regarding FP use. Though such decisions have direct impact on women’s health, few women (22.4%) used to decide on their own about using FP methods (Table 2). Women whose husbands were involved in decision-making regarding FP matters were nearly five times higher odds of using any type of contraception (OR: 4.6, 95% CI = 1.5–14.0). However, husband type of education did not significantly affect the use of contraception. Availability of enough human resource and easy accessibility of services by Muslim community were found to be the significant factors determining current FP use [OR: 0.03, 95% CI: 2.7 (1.1–7.1) and OR: 0.003, 95% CI: 0.3 (0.1–0.7), respectively].

**Table 2:** **Key decision influencers in family planning use among respondents (*n* = 160)**.

Key decision influencers in FP use	Ever use of FP methods		Total
	No	Yes	
Husband	70 (60.3)	46 (39.7)	116 (72.5)
Family members	12 (80.0)	3 (20.0)	15 (9.4)
Friends	1 (100.0)	0 (0.0)	1 (0.6)
Social norms	2 (100.0)	0 (0.0)	2 (1.3)
Self	23 (88.5)	3 (11.5)	26 (22.4)

*Figures in parenthesis indicate percentages*.

Respondents were asked whether they had experienced any adverse effects due to use of FP methods, where it was found that 48 (30.0%) had experienced any side effect; however it was not significantly associated with current use of contraception. Respondents who had not received any information regarding FP from any of the sources like television, radio, newspaper, health workers, pamphlets, friends, or relatives were less likely to use FP methods compared with those who have received information from at least one source (OR: 0.01, 95% CI: 0.004–0.04) (Table 3).

**Table 3:** **Association between selected variables and current FP use (*n* = 160)**.

Variables	Current use of contraception		*p*-Value	OR (95% CI)
	Yes	No		
**Husband involvement in FP-related decision-making**				
Yes	36 (31.0)	80 (69.0)	**0.001**	**4.6 (1.5–14.0)**
No	3 (6.8)	41 (93.2)
**Educational status of husband**				
Madarasa only	7 (24.1)	22 (75.9)	0.35	
Modern education only	10 (23.8)	32 (76.2)		
Both madarasa and modern education	6(15.4)	33 (84.6)		
Illiterate	16 (32.0)	34 (68.0)
**Plan for children in future**				
Yes	9 (10.8)	74 (89.2)	**<0.001**	**0.2 (0.1–0.4)**
No	30 (39.0)	47 (61.0)		
**Difficulty in using the word “Family planning”**				
No	34 (27.0)	92 (73.0)	0.14	2.1 (0.8-6.0)
Yes	5 (14.7)	29 (85.3)	
**Enough human resource to provide FP services**				
No	9 (42.9)	12 (57.1)	**0.03**	**2.7 (1.1–7.1)**
Yes	30 (21.6)	109 (78.4)		
**Accessibility to services by Muslim community**				
No	21 (18.1)	95 (81.9)	**0.003**	**0.3 (0.1–0.7)**
Yes	18 (40.9)	26 (59.1)		
**Educational level of the respondents**				
Illiterate	24 (25.3)	71 (74.7)	0.82	
Madarasa education only	12 (23.5)	39 (76.5)		
Modern education only	2 (33.3)	4 (66.7)		
Both madarasa and modern education	1 (12.5)	7 (87.5)		
**Number of children**				
Less than or equal to 3	13 (18.8)	56 (81.2)	0.16	0.6 (0.3–1.2)
More than 3	26 (28.6)	65 (71.4)		
**Occupation of the husband**				
Agriculture	13 (29.5)	31 (70.5)	0.39	
Labor	24 (24.0)	76 (76.0)		
Business	2 (12.5)	14 (87.5)		
**Occupation of the respondents**				
Agriculture	32 (23.0)	107 (77.0)	0.31	0.6 (0.2–1.6)
Others (labor + business)	7 (33.3)	14 (66.7)		
**Experienced adverse effects due to use of FP methods**				
No	26 (21.3)	96 (78.7)	0.11	0.5 (0.2–1.2)
Yes	13 (34.2)	25 (65.8)		
**Received FP information from at least one source**				
No	6 (5.0)	114 (95.0)	**<0.001**	**0.01 (0.004–0.04)**
Yes	33 (82.5)	7 (17.5)	

*Figures in parenthesis indicate percentages*.

*Figures in bold indicate significant at 95% CI*.

*OR, odds ratio (calculated for only two categories); CI, confidence interval*.

**Figure 1:**
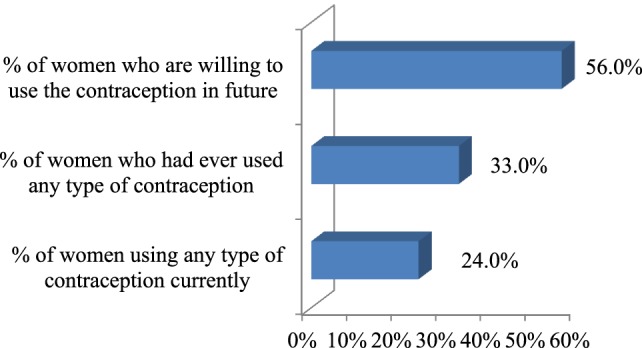
**Contraceptive use among the respondents**.

The percentage of respondents who had ever used contraception (32.5%) was found to be a bit higher than the percentage of respondents who had been using it currently (24.0%). Fifty-six percent women expressed desire to use FP methods in the future (Figure 1).

Women are afraid of Maulana (religious leader) and society, so don’t openly share about their FP use but are using secretly in an almost equal proportion to that of women of other ethnicities. [KII/FCHV/Maharajgunj]

Women come out of their houses with several excuses like shopping, medicines for child etc. and take pills or depo. But they tell that they don’t want their identity to be disclosed, thus we enter only codes. [consultative meeting/rep from Marie stopes, Family Planning Association of Nepal (FPAN), United Nations Population Fund (UNFPA) Nepal, etc.]

The number of FP users among Muslims was almost similar to that of women of other ethnicities; however, they still could not demand it actively due to societal norms.

It is of no use to go to health post, they never have depo-provera (injectables), so, I directly go to private medical shop and get depo with Rs 50 for one time. [Female FGD participant, Maharajgunj VDC]

Table 4 gives information about the most preferred methods of FP which were found to be injectables (Depo-Provera) followed by pills. Similarly, though FP services are provided free of cost in public health facilities, nearly half of the users sought services from private facilities.

Women prefer to take services from health centers far from their homes as they don’t want to be known by others. [consultative. meeting/Marie Stopes]

I use condom, but it’s not necessary to share this information with others [married man, FGD, Jahadi]

**Table 4:** **Different contraceptive methods being used by current users (*n* = 39)**.

Variables	Frequency	Percentage
**Types of modern FP methods**		
Condom	3	7.7
Pills	10	25.6
Injectables	20	51.3
Implant	2	5.1
Permanent method	4	10.3
**Source of FP methods**		
Government institution	22	56.4
Private institution	17	43.6

Secrecy about contraceptive use was their primary concern.

Table 5 shows that, out of 31 respondents who had discontinued FP use, most common reason for discontinuation was side effects related to FP methods like bleeding, body-ache, etc., accounting for nearly 42%.

I had heard that consumption of pills will lead to cancer. [female discussant at Tilaurakot]

One of my neighbors used to take Depo but it didn’t suit her [pause], there was heavy bleeding and she died because of that. (Eyes full of doubts) [Young female discussant, Tilaurakot]

**Table 5:** **Reasons behind discontinuing use of FP methods (*n* = 31)**.

Reasons	Frequency
Husband went abroad	4 (11.1)
Side effects	15 (41.7)
Lack of availability	5 (13.9)
Unknown about methods	1 (2.8)
Fear from social norms	5 (13.9)
Desire of children	1 (2.8)

*No. in parenthesis indicates percentages*.

Women expressed several myths and misconceptions regarding the FP use.

### Key Policy Designing Issues

If we direct our activities towards increasing the target number for Muslim FP users, figures (number of contraceptive users) will decrease even further [consultative meeting/representative, Local Non-government organizations (NGOs)]

Comprehensive service package with door to door approach can be an effective strategy for improving the uptake of FP services. [Health programme managers, consultative meeting]

If services are available at doorstep, then Muslim women don’t have to seek for health services without revealing their identity and definitely more women will use it. [Consultative Meeting/representative from UNFPA]

We need to meet target in the given time and where easy options are readily available, who will go for the difficult path. [consultative meeting/rep from FP-related NGOs].

FP should be advocated as means for family health not only for birth control. [consultative meeting, International Nepal Fellowship (INF) and Sakriya Sewa Samaj]

Both qualitative and quantitative findings illustrated that door to door service will be one of the effective strategy for improving the access to FP services and it will be better to provide services in comprehensive package for improving women’s health. (Table 6)

**Table 6:** **Key approaches to FP services according to respondents**.

Approaches to improve FP use	Yes	No

	Freq (%)	Freq (%)
Door to door service	95 (59.4)	65 (40.6)
Group focused program	66 (41.3)	94 (58.8)
Targeted program for Muslims	4 (2.5)	156 (97.5)
Awareness by Muslim leaders	3 (1.9)	157 (98.1)

From the consultative meeting held with different stakeholders working in the field of FP, it was found that use of the terminology “family planning” seemed to have created a barrier in FP use, as people felt that it was against their societal values. Respondents were more open to associating FP with increasing birth interval, than with practicing birth control. In place of the term “family planning,” some ideas were recommended by the community members include “Maa Jannat ki Dwar” (mother is the door to heaven), “Maa Jannat ki Chaabi” (mother is key to heaven), and “Khusahaal Jindagi” (happy life). However, in quantitative analysis, when women were asked whether they have difficulty in using the term “family planning,” out of 160 respondents, 34 (21.3%) expressed difficulty and among them, more than 85% were not using any contraceptives currently, but there was no any significant association.

## Discussion

This study has explored the existing situation of FP use in Muslim communities and has identified key policy-related issues, which, if dealt with adequately, while designing policy, will certainly help in the improvement of reproductive health indicators among women of marginalized communities.

Various policy documents reveal that it was first initiated as a limiting approach, which was later diverted toward a right-based approach and now a development approach. FP is considered central to the overall well being and development of a family, contributing toward improved health of women, more stable and prosperous families, as explained in several studies (13, 20). However, the rationale for FP is still propagated as a means to reduce family size to alleviate demographic and economic issues facing the country, which is seen by many citizens as an infringement to their personal decision-making process. This effect is particularly more pronounced in Muslim communities (21). Contemporary Muslim world is facing unprecedented challenges. With the entry into modern world, some have felt the need of FP and are using various measures, while, at the same time, there are certain groups that are still struggling to put forward their needs and desires in a restrictive social environment (21, 22). These contrasting views present an interesting opportunity for transformation, and interventions can be targeted to support the use of FP services for improved family health outcomes.

Similar to findings of another study done in Muslim communities (23), we found that sociocultural preference and unacceptability of contraception pose significant barriers to FP, despite strong interest of individuals to practice it. Nevertheless, while making inferences regarding unmet need, we should be also mindful that women were using FP secretly, and they chose not to reveal their identity. Thus, lower statistics of Muslim FP users even at the national level might be due to the reliance on records of supply of FP services at public sectors, whereas, Muslim women prefer private sector services due to greater privacy. Despite realization of needs of using FP methods openly, many are still struggling to use these methods secretly due to existing social values and institutions. Women do not want to disclose their identity for which they are paying for the services, which was otherwise being provided free of cost. This finding is supported by a similar study conducted in Muslim community, India (24).

Medroxyprogesterone (Depo-Provera) was the most commonly used FP methods (13), and it might be due to the fact that women seek for those methods which they can use on their own and is not known by others, so that they can keep their FP use status secret. Fear of side effects was identified as the most common reason for discontinuation of FP use, which was consistent with other studies (11, 13). Accessibility to services was found to be the significant factor affecting the use of FP, which highlights the importance of promoting the activities toward increasing access to services by all segments of population.

The principles of informed and voluntary decision-making are not new in sexual and reproductive health (SRH). They have been the fundamental tenets of quality FP services for decades but still individuals’ desire of using FP is facing opposition from prevailing social values and institutions (25). Issues of birth control are submerged within larger minefield of cultural polemics. Society has conflicting interpretation where one group exhibit vociferous antipathy to FP, while for another group contraception is overwhelmingly permissive (26). Given such variations in responses from different sections of society, development agencies should be careful to promote strategies and implement programs that are culturally sensitive and respectful of the religious priorities of the population. The study has identified several such strategies that can promote the ultimate goal of any FP program, i.e., improved family health and well-being, while being mindful of the religious and cultural ideals that prevail in a Muslim society.

Existing culture and societal values cannot be changed with blanket policies that set the same targets for all citizens. Blanket policies can either suppress certain cultures or devalue them by encouraging assimilation into a dominant culture. Policies need to be designed and implemented in such way that they do not contradict with existing social norms, and in doing so, include cultural rights and values of beneficiaries. Verses in the Qu’ran emphasize the importance of maintaining family harmony, and we need to extend the argument that if a family is large, tranquility in domestic life will be compromised (27). Rather than, advocating FP as a means to limit births, we need to deliver the message that it is an effective tool for overall family welfare. At the same time, religious interpretations should be focused in different way, that in Islam, God prioritizes having a healthy family over a plentiful family (13, 27).

Following are the main policy implications that can be derived from the study findings:

1. Religious leaders can be expected to interpret the religious text in the way of acceptability of new ideas and do not oppose to innovation, unless a new idea is perceived to contravene religious tenets, which is supported by other study as well (28). Religion does not denounce the use of FP, but the way it is interpreted is largely up to society (23).
2. Spousal communication, husband approval were found to be important determinants of the adoption of FP methods similar to Ethiopian study (29). Informed choice in FP is to empower women to make their own decisions regarding contraception, however still husband play dominant role in decision-making (25). As husband played significant role in choice and use of FP methods, involving husbands in any program intervention related to improved family health outcomes, therefore, may potentially be a high impact generating strategy for programmers. Hence, husband should be educated about responsible parenthood regarding family size, family health, decision-making, etc., where he should take decision considering the women’s preferences and health.
3. Information regarding the FP was found to be the significant factor associated with current use of contraception similar to other studies (13). Provision of information regarding the health benefits of the FP methods can be the potential strategy for improving the use of contraception among Muslim women (21).
4. There exists a substantial gap between women’s stated reproductive preferences and their use of contraception, corroborating findings from several other studies (29). Prevalence of contraceptive use obtained in our study was also relatively lower than that of national figures (49%), and these disparities in contraceptive use highlights the continuity of efforts for easy access to comprehensive, affordable, and high-quality FP information and services. Socio-cultural preference and unacceptability of contraception pose significant barriers to FP, despite strong interest of individuals to practice it; hence, use of contraceptives largely depend on their life circumstances. But the consequences of bearing children are solely borne by individual woman; thus, they resort to varied measures on their own to limit childbirth (30), however, privacy is their primary concern. Similar finding has been obtained in Indian study done among Muslims (24, 31).
5. Lower statistics of Muslim FP users even at the national level might be due to the reliance on records of supply of FP services at public sectors, whereas, Muslim women prefer private sector services due to greater privacy (24). Hence, this demands proper uniform registry and timely reporting from all sectors.
  Rather than pointing their cultural practices and social norms as barriers to FP service uptake, we should need to understand their values and create the policy which can provide women ample opportunity for best choice (e.g., good method mix, well-trained and empowered providers, good flow of contraceptive commodities). Misconceptions, if any, should be removed, and they should be allowed to take decision on their own provided that they have correct and right information. Policy should accommodate and respect individual’s cultural rights and voluntary FP is one of the most cost-effective investments a country can make in its future (32).
6. Rather than organizing mass campaigns and advocating FP as the way to limit births, emphasis on FP as a measure for birth spacing to protect the health of mothers and children would be more effective in improving its uptake. Furthermore, adoption of comprehensive package with FP and door to door approach for service delivery will definitely contribute to improve its accessibility and availability. Such comprehensive package will not only benefit women of marginalized community, but for all as well. Such policies will help to improve access and uptake of FP services in communities where it is generally believed that couple does not use contraceptives as they are grappled with inhibitions to adopt FP on religious grounds.

## Conclusion

This operational research explored the issue of FP in Muslim community, where it is still considered as taboo and provides in-depth understanding of policy gaps and key recommendations using mixed method approach. Individuals’ desire of using FP is facing opposition from prevailing social values and institutions. Society has conflicting interpretation where one group exhibit vociferous antipathy to FP, while for another group contraception is overwhelmingly permissive. Effective service delivery mechanism should be designed where FP program should not contradict the existing social values. Husband approval and secrecy of their personal identity affect use of any method of contraception. Future plan for children and prior information regarding FP found to affect current use of FP, significantly. FP word itself was found to be stigmatizing, so women prefer replacing the word FP with culturally appropriate one. It adds to the stock of literatures available in this topic and contributes to effective intervention planning and implementation. It provided the opportunity to inform the respondents about benefits of contraception and clear some of the myths and misconceptions regarding FP use as informal discussion were conducted after each interview and/or group discussion. Interviewing policymakers would add more valid information for the successes and failures of policies, but it was beyond the scope of this study. Being cross-sectional in nature, it could not establish the temporal relationship between the factors contributing to current FP use.

## Author Contributions

DS, TB, and SRA were involved in conception and design of the study. DS was involved in tool preparation. TB and SRA edited and finalized the tools. DS and VS were involved in data acquisition. DS, TB, SRA, and VS were involved in analysis and interpretation. All authors participate in report writing and providing intellectual input. DS and SRA prepared the draft which was critically reviewed and approved by all the authors. The manuscript has been read and approved by all the authors and the requirement for authorship was fulfilled by all authors.

## Conflict of Interest Statement

The authors declare that the research was conducted in the absence of any commercial or financial relationships that could be construed as a potential conflict of interest.
